# Preparation of Bioactive Titanium Surfaces via Fluoride and Fibronectin Retention

**DOI:** 10.1155/2012/290179

**Published:** 2012-11-08

**Authors:** Carlos Nelson Elias, Patricia Abdo Gravina, Costa e Silva Filho, Pedro Augusto de Paula Nascente

**Affiliations:** ^1^Biomaterials Laboratory, Instituto Militar de Engenharia, Pr Gen Tibúrcio 80, 22290-270 Rio de Janeiro, RJ, Brazil; ^2^Avenue Carlos Chagas Filho, 373, Cidade Universitária-21941-902 Rio de janeiro, RJ, Brazil; ^3^Rodovia Washington Luís km 235, 13565-905 São Carlos, SP, Brazil

## Abstract

*Statement of Problem*. The chemical or topographic modification of the dental implant surface can affect bone healing, promote accelerated osteogenesis, and increase bone-implant contact and bonding strength. *Objective*. In this work, the effects of dental implant surface treatment and fibronectin adsorption on the adhesion of osteoblasts were analyzed. *Materials and Methods*. Two titanium dental implants (Porous-acid etching and PorousNano-acid etching followed by fluoride ion modification) were characterized by high-resolution scanning electron microscopy, atomic force microscopy, and X-ray diffraction before and after the incorporation of human plasma fibronectin (FN). The objective was to investigate the biofunctionalization of these surfaces and examine their effects on the interaction with osteoblastic cells. *Results*. The evaluation techniques used showed that the Porous and PorousNano implants have similar microstructural characteristics. Spectrophotometry demonstrated similar levels of fibronectin adsorption on both surfaces (80%). The association indexes of osteoblastic cells in FN-treated samples were significantly higher than those in samples without FN. The radioactivity values associated with the same samples, expressed as counts per minute (cpm), suggested that FN incorporation is an important determinant of the *in vitro* cytocompatibility of the surfaces. *Conclusion*. The preparation of bioactive titanium surfaces via fluoride and FN retention proved to be a useful treatment to optimize and to accelerate the osseointegration process for dental implants.

## 1. Introduction

The phenomenon of endosseous implant osseointegration, conceptualized by Branemark as the “direct, structural and functional link between the living and orderly bone and the surface of an implant subjected to functional loads” [1], is fundamental to the success of dental implant applications. Commercially pure titanium (cp Ti) is the main material used for this purpose because it has good biocompatibility and adequate mechanical strength. Ti exposed to oxidizing agents spontaneously forms a 10-100 Å thick titanium oxide layer. This layer is stable in most media, especially under physiological conditions, and, surgically, it shows no change in thickness or corrosion. This ensures implant-bone tissue interaction and osseointegration [2]. The reactions of the tissue host with the biomaterial are determined by the surface properties of the biomaterial. The dental implant surface treatment should induce the differentiation of the desired cells [3]. Surface treatments of available implants promote changes in the mechanical, microstructural, and physical properties, as well as the wettability, energy, chemical composition, and density of chemical groups or molecules on the surface [2, 4].

This paper shows that the bone-implant interface strength is greater in dental implants with rough surfaces than in those with smooth surfaces [5, 6]. Treatments to increase the surface area for fibrin adhesion encourages implant adhesion. The presence of these surfaces also increases platelet activation, which produces large gradients of cytokines and growth factors through which leukocytes and osteogenic cells can penetrate the healing site [7]. Titanium surfaces coated with proteins can influence host reactions and thus enhance tissue integration [4]. Fibronectin is a major adhesion protein in the extracellular membrane, and it is important for cell adhesion, migration, proliferation, differentiation, and survival because it facilitates focal contacts with the receptors.

Appropriate changes in dental implant surface roughness can produce better anchoring strength and mechanical locking in the early stages of osseointegration [2, 6]. Moreover, surfaces with different microtopographies provide a larger area for fibrin adhesion, potentiate platelet activation, and favorably affect local angiogenesis and cellular functions including migration, alignment, orientation, attachment, and differentiation [5, 6].

Johansson et al. [8] observed that surfaces treated with fluoride are smoother than sandblasted surfaces but that fluoride-treated surfaces showed higher calcium-phosphorus binding capacity, which could indicate an increased ability of the surface to react with calcified tissues and promote integration between bone and implant. According to Ellingsen and Lyngstadaas [9], *in vitro* tests have shown that titanium fluoride treatments have a greater capacity for the nucleation of phosphate crystals than sandblasted Ti implants. *In vivo* fluoride ion-modified implants have generally proven superior to sandblasted surfaces in terms of osseointegration, ultimately increasing the removal torques.

Fibronectin is a major extracellular matrix protein that is known to promote cell attachment and spreading, differentiation, and phagocytosis. It is a dimeric glycoprotein found in all vertebrates in two basic forms: soluble (plasma and other fluids) and insoluble (extracellular matrix of various tissues). It has a molecular weight between 440 and 500 kDa. Disulfide bridges link one subunit to another via sites near the carboxy termini of each subunit. The fibronectin protein has folds that lead to structural remodeling and various conformations according to the medium [10–12].

Fibronectin (FN) functions in cell adhesion, migration, survival, proliferation, and differentiation as well as tissue organization. The FN molecule can interact with other biomolecules, such as collagen, proteoglycan, heparin, hyaluronic acid, fibrin/fibrinogen, plasmin, gangliosides, complement components, and also integral proteins of cell plasma membrane-integrins, as well as with itself [13].

Menezes [10] conducted a study to assess the interaction of human osteoblasts with films of human plasma fibronectin prepared under different pH conditions. The results showed no quantitative differences in the interaction of human osteoblastic cells (HOB) to different coatings, but qualitative differences were observed; osteoblasts adhered to each of the substrates in very different ways. The largest areas of cells adhesion were observed for substrates preincubated at 4.5 pH.

Petrie et al. [14] conducted a clinical study to evaluate the effects of specific bioactive coatings on the healing of bone tissue and the osseointegration of titanium dental implants. The author showed that surfaces containing a FN fragment for the integrin *α*5*β*1 (FNIII^7–10^) increase osteoblastic differentiation and optimize tissue formation and functional integration compared with untreated surfaces or surfaces containing only the RGD sequence.

The purpose of this study was to evaluate the effect of the fluoride treatment of cp titanium samples on the adhesion and proliferation of osteoblastic cells on surfaces with and without fibronectin coating.

## 2. Materials and Methods

### 2.1. Samples

Implants and discs of grade 4 machined cp Ti were provided by Conexão Sistemas de Prótese (Arujá, SP, Brazil). Samples were submitted to surface treatment and divided into four groups:

 Porous: samples treated in acidic solutions containing HNO_3_, H_2_SO_4_, and HCl (surface treatment similar to Porous implants available from Conexão Sistemas de Prótese);
 PorousNano: treatment similar to Group 1 followed by fluoride ion modification by immersion for one hour in a solution containing fluorine ions;
 Porous-FN: treatment similar to Group 1 with FN incorporation;
 PorousNano-FN: treatment similar to Group 2 with FN incorporation.

After treatments, samples from the Porous and PorousNano groups were washed with distilled water and absolute alcohol, dried in oven at 70°C for two hours, and packed and sterilized by gamma irradiation (25 kGy).

### 2.2. Surface Characterization

To characterize the surface morphology and identify differences in samples submitted to treatments with acids and/or fluorides, the samples were characterized by a high-resolution scanning electron microscopy (FEG/EDS, Philips XL30FEG). The results were complemented by analysis with an MFP-3D atomic force microscope (Asylum Research, CA, USA) operating in contact at room temperature mode. The cantilevers used were V shaped, NP-S model (Veeco Probes, CA, USA) with an 0.08 N/m spring constant, and calibrated using the thermal noise method. To reduce damage to samples and reduce noise, images were acquired using low-frequency scanning (1.0 Hz) with 256 × 256 pixel resolution. Image processing was performed in the program IGOR PRO (WaveMetrics, Portland, OR, USA) using a MFP-3D platform developed by Asylum Research.

### 2.3. Identification of Crystalline Phases

An X-ray diffractometer was used to identify crystalline phases on discs. X-ray diffraction for the analysis of thin films (grazing incidence technique) was conducted at 40 kV and 30 mA. A copper anode was used (Cu − K*α* = 1,542 Å) with an RU 200B model Rigaku generator and 0.02° step/minute.

### 2.4. Fibronectin Incorporation

Human serum fibronectin (Sigma-Aldrich Co., São Paulo, Brazil)) was diluted to 10 g/mL, pH 4.5 in previously filtered 20 mM sodium acetate (Reagen Laboratory Products, Paraná, Brazil) buffer solution. NaCl was added to the solution to maintain the medium's ionic strength between 0.145 and 0.150 mol·dm^−3^.

Samples from the Porous and PorousNano groups were coated with fibronectin at room temperature for 2 hours. Substrates with FN were washed with PBS (phosphate [0.01 M] buffered saline [0.15 M], pH 7.2) to remove nonadsorbed molecules. Then, the adsorbed molecules were detached using 0.1% trypsin and PBS. One to two minutes later, the excess was removed, and the resulting solution was collected and analyzed with a Spectrum 22PC spectrophotometer to quantify the adsorbed molecules. Spectrophotometry was also used to determine the FN's absorbance on both surfaces (protein concentration in solutions that absorb radiation). Negative (PBS) and positive (FN suspension 100 *μ*g/mL) controls were performed. The wavelength used was 550 nm (protein reading). 

### 2.5. Culture of Osteoblasts

Cells were maintained in polystyrene bottles containing DMEM (Dulbecco's Modified Eagle Medium) culture medium with low glucose, 10% fetal bovine serum (Soromed Industry, São Paulo, Brazil), and 1% essential amino acids solution (Minimum Essential amino acid solution 100x, Sigma-Aldrich) ascorbic acid (0.15 gL^−1^, Sigma-Aldrich) buffered with 10 mM HEPES (Sigma-Aldrich) and 14.3 mM NaHCO_3_ (Reagen). The pH of the medium was adjusted to 7.2. Cultures were incubated at 37°C in 5% CO_2_ atmosphere. The enzymatic cell detachment technique was used to transpose cells from the stock culture flask to substrates for the adhesion assay. Confluent cultures were treated with 0.2% trypsin (Difco Microbiology Co., USA) and 0.02% EDTA (Sigma-Aldrich) in saline solution (0.8% NaCl [Reagan], 0.01% KCl [Sigma-Aldrich]; 0.29% NaHPO_4_·7H_2_O [Reagan], and 0.02% KH_2_PO_4_ [Sigma-Aldrich] in H_2_O) for 5 minutes at 37°C. Then, the detached cells were collected, and the proteolytic action of trypsin was inhibited by adding fetal calf serum to the solution. The suspension was then centrifuged at 1500 rpm at 22°C, and the pelleted cells were resuspended in culture medium without fetal calf serum. The cell concentration/density of the suspension was estimated by counting in a hematimetric Neubauer chamber.

### 2.6. Interaction of Cells with Samples

After the cell concentration of the suspension was measured in a hematimetric chamber, 10^6^ cells/mL were taken and allowed to interact with the samples with and without FN coating, which totaled four groups. After an hour of interaction, the supernatants were discarded, and the cells that were attached (adsorbed and adhered) to surfaces were washed with PBS and fixed using glutaraldehyde (2.5% in PBS). Glutaraldehyde was used as fixative to avoid damaging cell integrity (glutaraldehyde contains two functional groups that link two proteins). This procedure was adopted because the use of formaldehyde (which has only one functional group) as a fixative profoundly deformed the cells. After fixation, cells were trypsinized and counted in a hematimetric Neubauer chamber. 

### 2.7. Surface Radioactivity

Human osteoblastic cells (HOBs) were cultivated to evaluate cell adhesion and proliferation by liquid scintillation counting. Cells from confluent HOB cultures were detached with trypsin, washed, and counted in a hematimetric chamber. Then, the culture was resuspended in DMEM containing serum and [^3^H]-thymidine (1143 cpm). After allowing incorporation for a period of 12 hours, the confluent cells were again detached and washed in DMEM without serum, and a liquid scintillator (Beckman, Rack III) was used to evaluate the radioactivity associated with cells. The resulting values were expressed as counts per minute (cpm). These cells, incorporating [^3^H]-thymidine, were associated with different surfaces (Porous, Porous-FN, PorousNano, and PorousNano-FN) for a period of 3 hours, and counts were carried out after 1, 2, and 3 hours. This cell behavior evaluation method allows accurate reproduction, favoring the future applicability of FN incorporation onto surfaces of dental implants.

## 3. Results

### 3.1. Surface Morphology

**Figure 1:**
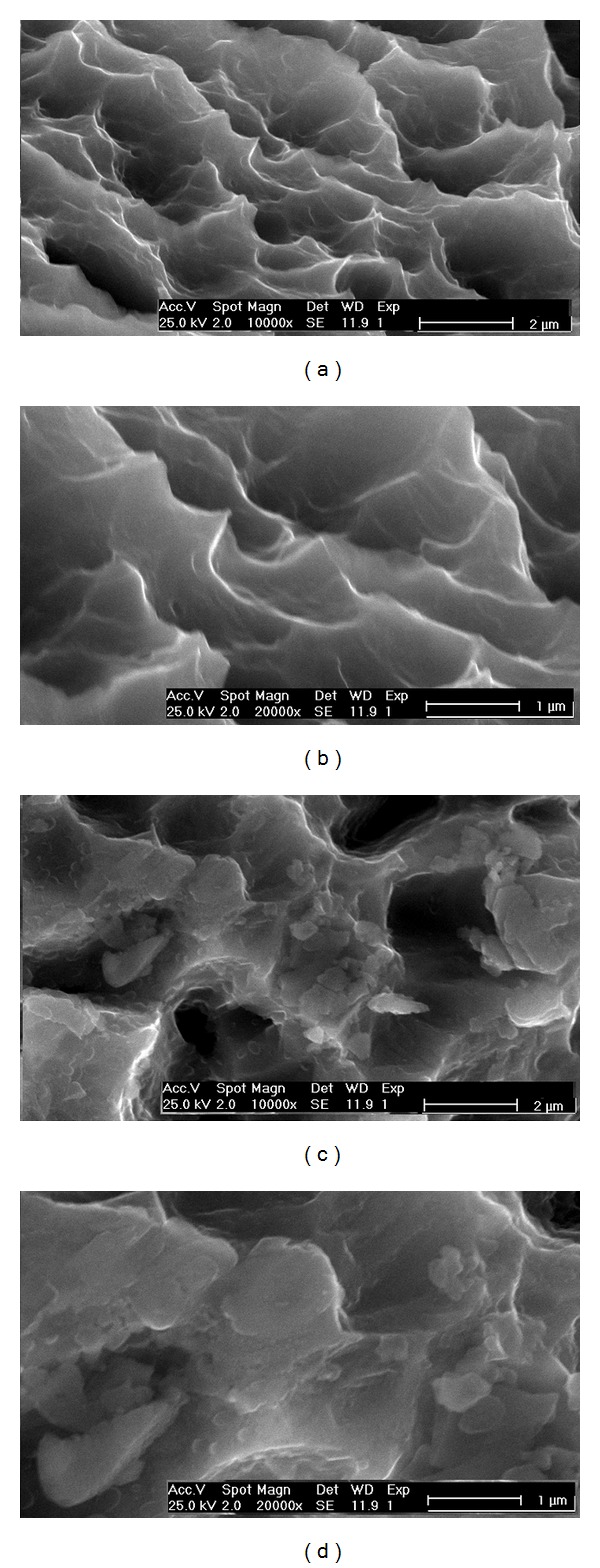
SEM images of the samples before coating with fibronectin. (a) and (b) Porous samples (acid treatment). (c) and (d) PorousNano samples (acid treatment followed by fluoride ion modification).

Figure 1 shows Porous and PorousNano titanium surfaces before coating with fibronectin. These surfaces exhibited microcavities with different sizes and sharp edges. Immersion into a solution containing fluoride ions (PorousNano) did not change the microcavity morphology, and the sharp edges persisted. A minor modification caused by immersion is shown in Figure 1(c); some white regions are observed when compared with Figure 1(b). At high magnification (Figure 1(d)), the PorousNano group showed evidence of particle clusters at the surface due to immersion in the solution containing fluoride ions. This is the major ultrastructural characteristic of the PorousNano sample.

**Figure 2:**
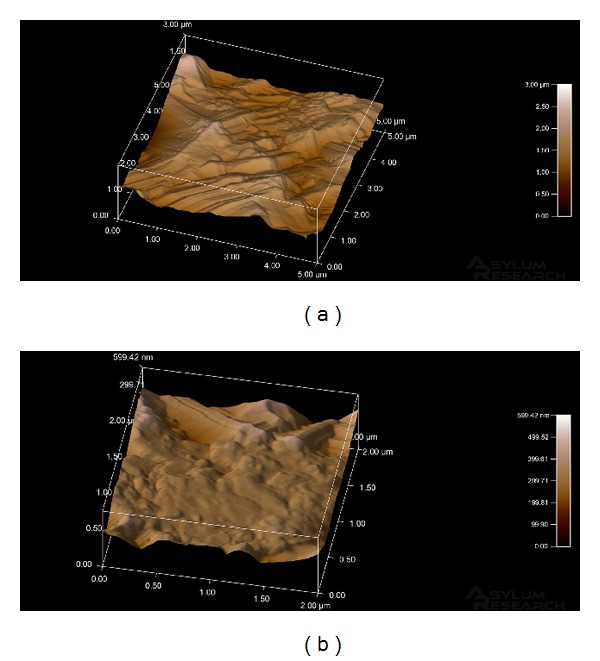
AFM images: (a) Porous and (b) PorousNano.

Figure 2 shows images obtained by atomic force microscopy. In Figure 2(a), the microcavity edges are more flattened but maintain the sharp features that seem to assist or facilitate the adsorption of fibronectin and cells.  Figure 2(b) shows the PorousNano sample surface at high magnification, demonstrating that the roughness pattern at the microcavity edges is flattened by immersion treatment in a solution containing fluoride ions.

The surface roughness of the Porous sample (Figure 2(a)) was 1759.7 nm (±204.4 nm), whereas the roughness of the PorousNano surface sample (Figure 2(b)) was 1406.5 nm (±226.9 nm).

### 3.2. Identification of Crystalline Phases

**Figure 3:**
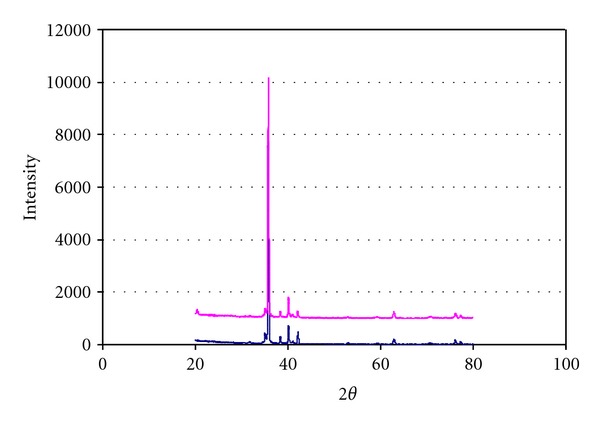
X-ray diffraction spectra of Porous and PorousNano surfaces.

Figure 3 shows the X-ray diffraction spectra of the Porous and PorousNano surfaces. Both contain only titanium as the crystalline phase.

### 3.3. Incorporation of Fibronectin

Porous and PorousNano titanium surfaces were treated with crystal violet (1% in PBS), and the stain associated with the surfaces was eluted with methanol. Negative (buffer solution) and positive (FN suspension) controls were assessed by spectrophotometry. The absorbance was proportional to the amount of the cells such that more the cells on the surface corresponded to larger absorbance values. Cells treated with PBS measured at 0.326 absorbance units (AU) at 550 nm (reading for proteins). The FN suspension (100 *μ*g/mL) measured at 2.992 units. After the FN incorporation in Porous, and PorousNano tablets, both spectrophotometric measurements were 2.473 absorbance units (82.6%) at 550 nm, indicating that the two surfaces exhibit similar behavior with respect to fibronectin incorporation.

### 3.4. Interaction of Cells with Surfaces

A total of 10^6^ human osteoblastic cells/mL were delivered to Porous and PorousNano surfaces, and, after a 1.0 hour interaction, 7.9 × 10^4^ cells/mL and 2.3 × 10^5^ cells/mL were associated with the Porous and PorousNano (no protein coating) surfaces, respectively. The combination of cells to both surfaces, with and without the fibronectin incorporation, resulted in different association indices. For comparison, association index values were considered null for samples without FN. After one hour, the association indices values of cells with samples with FN showed increase of 44.7% (±0.8%) and 57.4% (±0.3%) for Porous-FN and PorousNano-FN surfaces, respectively, compared to the same surfaces without FN.

The cell-surface interaction index of the PorousNano-FN was approximately 28% higher than that of the Porous-FN.

### 3.5. Surface Radioactivity

**Figure 4:**
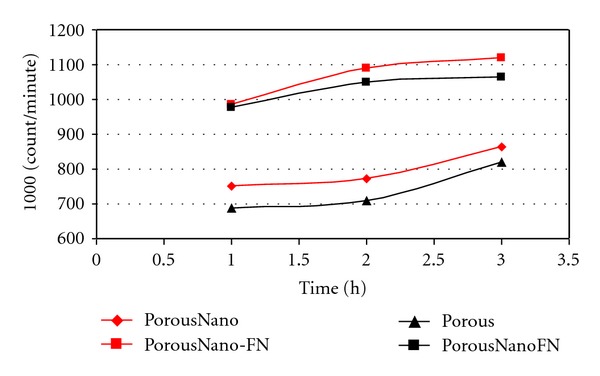
Radioactivity associated with osteoblasts on Porous, Porous-FN, PorousNano, and PorousNano-FN surfaces. The resulting values were expressed as counts per minute (cpm).

After the incorporation of [^3^H]-thymidine for 12 hours, the radioactivity associated with osteoblast cells was evaluated. Subsequently, 1.8 × 10^6^ cells/mL, corresponding to 1,100 cpm, were delivered to Porous, Porous-FN, PorousNano, and PorousNano-FN surfaces. The results are shown in Figure 4.

After one hour of interaction, 70% of cells (0.751 cpm) were associated with the PorousNano surface. This number is most likely low because some cells died or were not associated with the sample at the beginning of the process. The number of associated cells increased with interaction time, reaching 0.864 cpm after three hours; that is, there was a 15% increase in the amount of associated cells due to proliferation and cell division.

Only 64% of the cells interacting with the Porous surface (0.687 cpm) remained associated after one hour, but this number increased approximately 32% after 3 hours of interaction, reaching 0.905 cpm.

On the Porous-FN surface, 90% of cells (0.976 cpm) were associated after 1 hour of interaction. The number of attached cells increased 9% after three hours, reaching 1.064 cpm. For the PorousNano-FN surface, 92% of cells (0.986 cpm) were associated after one hour of interaction, and this number increased by 11.5% over three hours, reaching 1.100 cpm. 

## 4. Discussion

Figure 1(a) shows the surface morphology of a Porous sample obtained by immersion treatment in acid solution. The acid etching produces a homogeneous surface characterized by microcavities surrounded by tapered summits. This pattern of roughness produces a homogeneous surface without preferential roughness orientation.

Figure 1(c) shows that the immersion of the Porous surface in a solution containing fluoride ions did not change the microcavity morphology, and the sharp edges persisted. At higher magnification, the presence of flatter areas and smaller micropeaks may be noted although these surfaces remain tapered. This change may be associated with the high reactivity of fluorine ions and the chemical susceptibility of titanium oxide to these ions, which may produce a coalescence of peaks. These results are consistent with those of Ellingsen and Lyngstadaas [9] and Johansson et al. [8], which showed that titanium surfaces treated with fluoride present smoother microtopographies and lower *R*_*a*_ values than acid-treated surfaces without fluoride. Figure 1(d) demonstrates the presence of microcavities, summits, and conglomerates on their edges, most likely due to the corrosion process and consequent decrease in surface roughness for the surface subjected to immersion in solution containing fluoride.

Images obtained by atomic force microscopy (Figure 2) show that both the Porous and PorousNano surfaces exhibit microcavities surrounded by summits. Like the high-resolution SEM images, the AFM images indicate that summits and microcavities of the PorousNano sample surface have smoother edges although they remain tapered. These sharp edges seem to assist or facilitate the adsorption of FN and cells.

As measured based on the images obtained through AFM, the roughness of the PorousNano surface sample was lower than that of the Porous surface, demonstrating that treatment with fluoride reduced the summit height, most likely due to the reaction of titanium oxide with fluoride ions. This ultrastructural aspect of the summits contributes to the more homogeneous roughness pattern of the PorousNano surface, in addition to the presence of smoother areas and larger microcavities.

The presence of only one crystalline phase of titanium was revealed by X-ray diffraction of the Porous and PorousNano samples. It is likely that the immersion in a solution containing fluoride ions adds only a small amount of this element to the titanium surface and that this trace amount of fluoride cannot be detected by the XRD technique for the analysis of thin films (grazing incidence technique).

Approximately 80% of the FN allowed to interact with Porous and PorousNano surfaces was adsorbed (2.473 AU). This result demonstrates that the chemical treatment with acids (Porous) and chemical treatment with acids followed by immersion in solution containing fluoride ions (PorousNano) did not affect the incorporation of biomolecule; that is, the presence of the fluoride ion did not influence the protein adsorption. Dos Santos et al. [15] observed that FN adsorption to anodized titanium samples was 68%. It can be concluded that titanium surfaces have an affinity for fibronectin and that differences in the percentage of incorporation in different studies most likely are due to the conditions under which the FN was reacted with the surfaces (pH used, for example) and/or the various treatments performed on them.

Cell counting in a hematimetric chamber is a sensitive and accurate technique for the evaluation of cell adhesion to titanium surfaces. In this study, the PorousNano surface showed a stronger association with osteoblastic cells (2,3 × 10^5^ cells/mL) than the Porous surface (7,9 × 10^4^ cells/mL) after one hour of interaction. Because 10^6^ cells/mL were taken to interact with surfaces, approximately 8% adhered to the Porous sample, while 23% were associated with the PorousNano sample. These indices suggest that the surface subjected to chemical treatment followed by immersion in a solution containing fluoride ions favors the adhesion of most cells during the initial interaction period. As mentioned earlier, the association indices of the Porous and PorousNano surfaces without fibronectin were considered null for evaluations of the influence of protein on cell behavior. Thus, the number of cells associated with the PorousNano with FN surface increased 57.4% compared with the same surface without the biological variable. For the Porous with FN surface, the increase in cell adhesion was 44.7% compared to the same area without the protein. These indices show that the protein variable is responsible for the significant increase in the number of cells attached to the surfaces, confirming the results of Ku et al. [16], who also reported an increase in the adhesion rate of cells to surfaces treated with recombinant fibronectin. They showed that, for TiO_2_, cell adhesion was initiated after 3 hours and had significantly lower cell numbers for all measurement points compared with FN. The present work showed the same results.

The cell-surface interaction index of PorousNano with FN was approximately 28% higher than that of the Porous with FN surface. This study demonstrates that, among the four types of surfaces examined, the PorousNano with fibronectin coating most favors the adsorption and adhesion of osteoblastic cells during the tested interaction period. In addition, the study provides strong evidence that FN incorporation into titanium surfaces is much more relevant for biocompatibility and the consequent acceleration of the osseointegration process than surface treatment with acid and/or immersion in solution containing fluoride ions.

A total of 1.8 × 10^6^ cells/mL (1.100 cpm) were allowed to interact with Porous, Porous-FN, PorousNano, and PorousNano-FN titanium surfaces for three hours. After one hour of interaction, 92% of the cells were associated with the PorousNano-FN surface, and 90% of cells were associated with the Porous-FN surface, while 70% and 64% of cells were associated with the PorousNano and Porous surfaces (without FN), respectively. These results confirm that the protein coating accelerated the adsorption of cells during the initial interaction period (adaptation period). This can be explained by the fact that when fibronectin is allowed to interact with titanium samples under ideal conditions of pH such that its cryptic sites are exposed, the fibronectin signals to osteoblasts to activate the cell cycle and initiate the secretion of ECM proteins. 

The Porous-FN and PorousNano-FN surfaces showed similar behavior during the three-hour interaction, both during the initial adherence of cells (approximately 90% for both surfaces) and in their proliferation. The cell number increased by 14% for the sample PorousNano-FN and 12% for Porous-FN in the first 3 hours of interaction (Figure 4). Ku et al. [16] also demonstrated that the biomimetization of titanium surfaces with fibronectin increased the adhesion, proliferation, and differentiation rates of cells.

In samples without FN, this study showed that within one-to-three hours of interaction, the number of cells attached to the PorousNano surface increased by 32%, while the number of cells attached to the Porous surface increased by 15%. This difference shows that the surface that received acid treatment followed by immersion in a solution containing fluoride ions (Nano) showed accelerated cell division and proliferation compared to the Porous surface. Figure 4 shows that the PorousNano surface without fibronectin coating exhibited the greatest increase in cpm as a function of time over 3 hours. Ellingsen and Lyngstadaas [9] and Johansson et al. [8] showed that fluoride-treated surfaces have a greater capacity to react with biological tissues and nuclear phosphate crystals *in vitro*, in addition to offering greater osseointegration resistance *in vivo*. Although previous studies have used different methodologies for fluoride treatment, their results also suggest that the presence of fluoride ions on titanium surfaces facilitates various osseointegration processes. Analysis of the experimental cell adhesion and proliferation data presented in Figure 4 showed that the cell behavior was similar in all samples containing fibronectin. The results of this study show that the FN is critical to the biocompatibility of surfaces of titanium implants, but when this protein is not present, treatment with acids and fluorides seems to favor more tissue integration than treatment with acid only (i.e., no fluoride).

## 5. Conclusions

Based on the experimental results, it can be concluded that

- the surfaces of titanium samples treated with fluoride ions (PorousNano) retained the basic microstructural characteristics of surfaces not treated with fluoride (Porous),
- the Porous and PorousNano surfaces incorporated similar levels of FN (approximately 80%) over the time tested (3 hours), demonstrating that the presence of fluoride ions did not influence protein adsorption,
- the association indices of HOB cells to the four tested surfaces suggest that FN incorporation is critical for the *in vitro* cytocompatibility of surfaces,
- FN-treated samples showed significantly higher percentages of associated cells during the initial period of one hour, confirming that FN (the biological variable) had a greater effect on the adhesion and proliferation of cells than the fluoride treatment of titanium surfaces used in this study.
